# Integrative Single-Cell Transcriptomic Analysis Identifies Candidate Endothelial Activation Signatures in Atopic Dermatitis

**DOI:** 10.3390/cimb48101039

**Published:** 2026-10-07

**Authors:** Fang Cao, Hui Zhang, Cui Guo, Jing Guo

**Affiliations:** School of Clinical Medicine, Chengdu University of Traditional Chinese Medicine, Chengdu 610072, China; caofang@cdutcm.edu.cn (F.C.); zhanghui@stu.cdutcm.edu.cn (H.Z.)

**Keywords:** atopic dermatitis, endothelial cells, hdWGCNA, pseudotime trajectory, machine learning, SHAP

## Abstract

**Background**: Endothelial activation is a recognized histological feature of atopic dermatitis (AD), but its transcriptional programs remain incompletely characterized. **Methods**: We reanalyzed public single-cell RNA-seq data from the skin of five patients with AD and four healthy donors using AD-associated gene set scoring, CellChat, endothelial hdWGCNA and pseudotime ordering, and examined candidate genes with eight machine learning classifiers and SHAP in public bulk transcriptomes of lesional and non-lesional AD skin. **Results**: Inferred intercellular interactions were more numerous in AD (1348 vs. 1111), with COLLAGEN, CD99 and MIF showing the largest gains in communication strength. Differences in AD-associated gene set scores between AD and healthy endothelial cells were small and inconsistent between scoring approaches. Among seven endothelial modules, Endo7, an NF-κB-responsive immediate-early module containing SELE, ICAM1, IRF1 and NFKBIA, overlapped most strongly with the AD gene set (10 genes; approximately 30-fold enrichment). AD endothelial cells were concentrated at intermediate pseudotime positions (Cohen’s d = 0.334). In bulk skin, classifiers based on 14 LASSO-selected genes separated lesional from non-lesional AD skin in a small test set (AUC 0.70–0.98; likely optimistic estimates, as feature selection preceded the training–test split and paired samples from the same patients may span both partitions), with SELE, TOP2A and IRF1 ranked highest by SHAP. **Conclusions**: This hypothesis-generating analysis identifies AD-associated endothelial activation signatures and candidate genes that require confirmation in independent, clinically characterized cohorts and experimental studies.

## 1. Introduction

Atopic dermatitis (AD) is among the most prevalent chronic inflammatory diseases, affecting approximately 20% of children and 7–10% of adults in high-income countries, and its global burden has grown steadily over the past three decades [1,2]. The clinical picture—persistent pruritus, barrier-disrupted eczematous skin, and recurrent bacterial and viral superinfections—is disabling, yet it has also been instructive, directing investigators toward a core mechanistic axis: epidermal barrier dysfunction enables allergen penetration, which drives a type 2 immune response dominated by IL-4 and IL-13 [3]. This framework has yielded genuine therapeutic progress. Dupilumab, which blocks the shared IL-4Rα receptor, has substantially improved the management of moderate-to-severe AD [4,5]. In the LIBERTY AD CHRONOS trial, 64% and 69% of adults treated with dupilumab weekly or every 2 weeks, respectively, together with topical corticosteroids achieved at least a 75% improvement in the Eczema Area and Severity Index (EASI-75) at week 16, compared with 23% of those receiving placebo plus topical corticosteroids [6]. Nevertheless, some patients respond only partially or relapse after treatment withdrawal [7,8], and chronic lesions show additional Th1 and Th22/Th17 activation superimposed on persistent type 2 inflammation [9,10]. Beyond biologics, agents acting on inflammatory and oxidative stress pathways, including plant-derived flavonoids and repurposed drugs such as ezetimibe, have shown anti-inflammatory activity in chemically induced AD models [11,12]. Incomplete responses and relapse motivate the search for additional disease-associated cellular programs, including those of the dermal vasculature.

The vasculature has been visible in the lesional microenvironment for decades, yet has received comparatively little systematic mechanistic investigation. Classical histopathological studies of AD described perivascular lymphocytic infiltrates around the superficial dermal venules, accompanied by endothelial cell hypertrophy and thickening of the venular basement membrane [13], and dense perivascular infiltrates remain a characteristic feature of lesional skin [14]. E-selectin (SELE) and vascular cell adhesion molecule 1 (VCAM1) are expressed on cytokine-activated endothelium and mediate leukocyte rolling and firm adhesion, respectively [15,16]. In AD lesions, increased endothelial E-selectin expression has been reported consistently [17,18], whereas findings for VCAM-1 differ between studies: endothelial VCAM-1 expression was described in early immunohistochemical work [17], but no endothelial VCAM-1 signal was detected in a recent analysis of AD lesions [18]. Elevated serum soluble E-selectin has also been shown to correlate with disease severity in independent patient cohorts [19,20]. These observations are usually interpreted as endothelial responses to cytokines produced by T cells and keratinocytes [21]. Cytokine responsiveness and an active contribution to leukocyte recruitment are not mutually exclusive, and the transcriptional programs that accompany endothelial activation in AD skin cannot be resolved by histology or bulk transcriptomics alone.

Single-cell RNA sequencing (scRNA-seq) enables simultaneous profiling of individual cell-type transcriptomes in lesional skin. Previous single-cell studies of AD have characterized keratinocyte, fibroblast and immune-cell heterogeneity [22,23], and Reynolds et al. showed, with in situ validation, that prenatal vascular endothelial cell programs re-emerge in inflammatory skin diseases, including AD [24]. Spatial transcriptomics combined with scRNA-seq has further mapped the inflammatory cell network of AD lesions [23], and multi-omics approaches continue to refine current models of AD pathogenesis [25]. Less is known about how AD-associated genes are distributed across endothelial transcriptional states, how endothelial cells are positioned within inferred intercellular communication networks, and which endothelial co-expression programs accompany activation in AD skin. Scoring single cells against a curated set of AD-associated genes indicates which cell populations show higher expression of these genes. Network-level approaches, including intercellular communication inference, co-expression module analysis and pseudotime ordering, further allow endothelial activation to be examined as a structured program rather than as a collection of individually changed genes.

In this exploratory study, we reanalyzed publicly available scRNA-seq data from the skin of five patients with AD and four healthy donors, together with a public bulk transcriptomic dataset of lesional and non-lesional AD skin. Using an AD-associated gene set, CellChat intercellular communication inference, hdWGCNA co-expression network analysis and pseudotime ordering, and machine learning classifiers with SHAP interpretation, we aimed to characterize AD-associated endothelial transcriptional programs, to examine the position of endothelial cells within inferred intercellular communication networks, and to prioritize candidate genes for future experimental and clinical validation.

## 2. Materials and Methods

### 2.1. Single-Cell RNA Sequencing Data Processing and Integration

Publicly available scRNA-seq data from skin biopsies of patients with AD and healthy donors were retrieved from the Gene Expression Omnibus [26] (accession GSE269981), where they had been deposited by Chennareddy et al. [27]. The series comprises skin biopsy samples from five patients with AD and four healthy controls, profiled on the 10x Genomics platform, and all nine samples were analyzed. The groups were not matched by design. The GEO records do not provide individual-level age, sex, disease severity, disease duration or treatment exposure, and these characteristics could not be assigned to individual samples; the available sample information is summarized in Supplementary Table S1. Raw count matrices underwent quality control in Seurat (v4.3.0) [28,29]: cells with fewer than 200 or more than 6000 detected genes, or with mitochondrial transcript fractions exceeding 20%, were excluded. Doublets were identified and removed using DoubletFinder (v2.0) [30,31]. Library size normalization (NormalizeData) and highly variable gene selection (n = 3000; FindVariableFeatures) preceded scaling and PCA. Batch effects across donors were corrected with Harmony (v0.1.1) [32,33], after which the top 30 harmonized principal components were used for UMAP embedding and graph-based clustering (FindNeighbors; FindClusters at resolution 0.5). Cell type annotation relied on canonical marker genes curated from the literature: KRT14/KRT5 for keratinocytes, PECAM1/VWF for endothelial cells, COL1A1/DCN for fibroblasts, CD68/LYZ for macrophages, TYRP1/DCT for melanocytes, and CD3D/CD3E for T cells. Cluster marker genes were confirmed with FindAllMarkers (Wilcoxon test, min.pct = 0.25, log2FC threshold = 0.25) [34].

### 2.2. Cell–Cell Interaction Analysis

Intercellular communication networks were inferred using CellChat (v1.6.1) [35], applied independently to the AD and healthy subsets of the integrated dataset. CellChat scores ligand–receptor interactions based on the law of mass action, incorporating receptor and ligand expression levels and known cofactor participation. Differential interaction analysis between conditions was performed using the compareInteractions and netVisual_diffInteraction functions [36]. The number of inferred interactions and the aggregate interaction strength (the sum of the communication probabilities of all inferred interactions) were compared between conditions, and pathways were ranked by the difference in communication strength; the top 20 are reported. Networks were inferred from cells pooled across donors, so donor-to-donor variability and unequal cell numbers between conditions were not modeled, and between-condition differences are reported descriptively. Chord diagrams for selected pathways were generated with the netVisual_chord_gene function.

### 2.3. AD-Associated Gene Set Construction and Scoring

The AD-associated gene set (n = 134; Supplementary Table S2) was assembled in an earlier network pharmacology phase of this project. AD-related genes retrieved from the GeneCards, OMIM and DisGeNET databases were intersected with the predicted protein targets of candidate phytochemicals obtained from TCMSP, SwissTargetPrediction and SuperPred, and genes present in both lists were retained. The resulting set comprises AD-related genes that are also annotated as potential pharmacological targets; no genes were selected on the basis of genetic association data. Of the 134 genes, 37 were present in the expression matrix used for scoring and were retained for single-cell scoring (the remaining 97 genes were absent from the processed expression matrix). Scoring was restricted to [CONFIRM: the 3000 highly variable genes retained in the processed expression matrix rather than the full gene space], so the 97 absent genes were not scored; their absence, therefore, reflects the gene space of this matrix and not necessarily a lack of expression in skin, and the scores represent only the 37-gene subset. Single-cell gene set scores were calculated using Seurat’s AddModuleScore function [37], which compares the mean expression of target genes against a background of randomly sampled control gene sets of equal size and expression-bin composition. Score distributions were compared between conditions using Wilcoxon rank-sum tests with Bonferroni correction for multiple cell-type comparisons; as these tests treat individual cells as observations, the resulting *p* values are reported descriptively.

### 2.4. hdWGCNA of Endothelial Cells

Endothelial cells were isolated from the integrated Seurat object and re-processed for hdWGCNA (v0.2.15) [38,39]. Metacells were constructed by aggregating the transcriptional profiles of k = 25 nearest neighbors per cell using the ConstructMetacells function, reducing technical noise while preserving biological variation. Soft power thresholding was performed across powers 1–30, and the lowest power yielding a scale-free topology model fit R^2^ ≥ 0.85 was selected (β = 6). Co-expression modules were constructed with blockwiseConsensusModules (mergeCutHeight = 0.25, minModuleSize = 50) [40]. Module eigengene (ME) values and module membership (kME) scores were computed for all genes; within each module, genes were ranked by kME, and the top-ranked genes were designated hub genes. The top 100 genes of each module with their kME values are provided in Supplementary Table S3. Functional annotation of Endo7 was performed using Enrichr [41] with the top 100 Endo7 hub genes as input and Enrichr’s default background gene set, against the GO Biological Process, Cellular Component and Molecular Function 2023 and KEGG 2021 Human libraries; terms with Benjamini–Hochberg adjusted *p* < 0.05 were retained.

### 2.5. Target Gene–Module Enrichment and Pseudotime Trajectory Analysis

To test whether AD-associated genes were over-represented in individual endothelial co-expression modules, the 37 scored genes of the AD-associated gene set were intersected with the gene membership lists of each hdWGCNA module. Fold enrichment was calculated as the ratio of observed to expected overlap, where the expected overlap was derived from the module size relative to the total number of expressed genes. Statistical significance was assessed using a one-sided hypergeometric test, with adjusted *p*-values computed by the Benjamini–Hochberg procedure. Overlap counts, fold enrichment and adjusted *p* values for each module are shown in later. Score distributions for overlapping target genes were additionally compared between AD and healthy endothelial cells using the Wilcoxon rank-sum test at the cell level.

To explore the ordering of endothelial transcriptional states, pseudotime trajectory analysis was performed with Monocle 2 (monocle R package R 4.5.0) [42,43] on the subsetted endothelial population. Dimensionality reduction was performed with the DDRTree method (reduceDimension), and cells were ordered along the learned principal graph with orderCells, which also partitions the graph into discrete cell states. Because pseudotime has no intrinsic direction, the root was set to the state containing the highest proportion of cells from healthy donors (state 1), on the assumption that healthy endothelium represents the baseline condition. Because this choice determines the direction of the ordering, pseudotime is interpreted as an inferred ordering of transcriptional states. Differences in pseudotime distribution between AD and healthy endothelial cells were tested using the Wilcoxon rank-sum test at the cell level, and effect size is reported as Cohen’s d. The expression of individual AD-associated genes along pseudotime was visualized using locally smoothed regression curves stratified by group, and module eigengene scores for each of the seven hdWGCNA modules were similarly projected onto the pseudotime axis.

### 2.6. Machine Learning Classification and SHAP Interpretation

To examine whether candidate genes also distinguish lesional AD skin at the tissue level, we analyzed a public bulk RNA-seq dataset of skin biopsies from patients with AD (GEO accession GSE121212) [44], comprising lesional (n = 20) and non-lesional (n = 27) samples; 20 patients contributed both a lesional and a non-lesional sample. Genes from the AD-associated gene set that differed significantly between lesional and non-lesional samples were used as candidate features. Non-negative matrix factorization (NMF) [45,46,47] was applied to the lesional samples to explore molecular subtypes, with the optimal rank determined by cophenetic correlation and dispersion coefficients across ranks 2–10. LASSO logistic regression [48,49] with cross-validated selection of the penalty parameter was used to select a reduced feature set. Samples were then randomly divided into a training set (70%; n = 33) and a held-out test set (30%; n = 14; 6 lesional and 8 non-lesional samples). Eight supervised classifiers were trained on the LASSO-selected features in the training set: Partial Least Squares discriminant analysis (PLS-DA), Random Forest (RF), Decision Tree (DTS), Support Vector Machine with radial kernel (SVM), Logistic Regression, K-Nearest Neighbors (KNN), Neural Network (single hidden layer), and Gradient Boosting (glmBoost). All models were implemented in the caret R package (v6.0-94) [50,51] with hyperparameters tuned by 10-fold cross-validation repeated five times within the training set. Performance was evaluated on the held-out test set by AUC, accuracy, sensitivity, precision, F1 score, Cohen’s Kappa, and Matthews Correlation Coefficient (MCC). LASSO selection was performed on the full dataset before the split, and samples were allocated at random without grouping by patient. SHAP values [52] were computed for the logistic regression model, which had the highest test-set AUC, using the DALEX package (v2.4.3) [53,54] and were visualized with plotting functions from the SHAPforxgboost package 0.2.0 [55]. The explained output was the predicted probability that a sample was lesional, and the expected value E[f(x)] corresponds to the mean predicted probability in the training set. Feature importance was summarized as the mean absolute SHAP value, and waterfall, beeswarm, force and dependence plots were generated to visualize individual feature contributions.

## 3. Results

### 3.1. Single-Cell Transcriptomic Profiling of AD Skin

**Figure 1:**
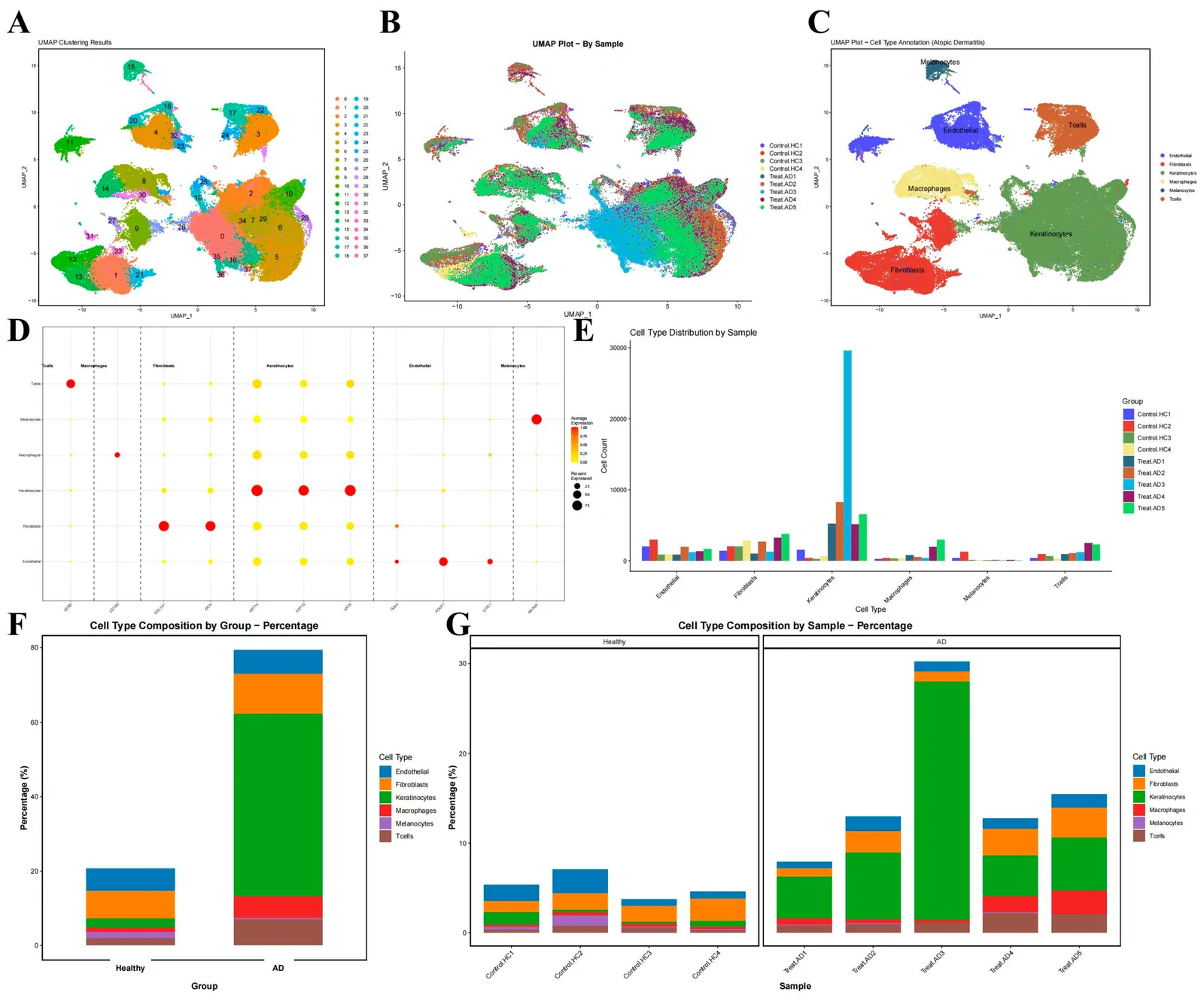
Single-cell transcriptomic atlas of atopic dermatitis skin. (**A**) UMAP visualization of 38 cell clusters from the integrated scRNA-seq analysis. (**B**) UMAP colored by sample of origin, showing integration across nine donors (five AD, four healthy). (**C**) Cell type annotation UMAP identifying six major populations: endothelial cells, fibroblasts, keratinocytes, macrophages, melanocytes, and T cells. (**D**) Dot plot of canonical marker gene expression across annotated cell types. (**E**) Bar chart of absolute cell type counts stratified by individual sample. (**F**) Stacked bar chart of cell type composition by group (healthy vs. AD), expressed as a percentage of all cells analyzed from both groups. (**G**) Cell-type composition of each individual sample, expressed as a percentage of all cells analyzed; the relative heights of the segments within each bar show the within-sample composition.

After quality filtering, doublet removal, and Harmony-based batch correction, the integrated dataset comprised cells from five AD patients (Treat_AD1–AD5) and four healthy controls (Control_HC1–HC4) distributed across 38 UMAP clusters, which were annotated into six major cell types: keratinocytes, endothelial cells, fibroblasts, macrophages, melanocytes, and T cells (Figure 1A–C). Sample mixing within UMAP space was satisfactory across all donors (Figure 1B), with no cluster showing exclusive composition from a single individual, supporting the validity of cross-condition comparisons.

Cell-type proportions differed between conditions (Figure 1E–G). In Figure 1F, the number of cells of each type is expressed as a percentage of all cells analyzed from both groups, so bar heights also reflect the larger number of cells recovered from AD samples. Within individual samples (Figure 1G), keratinocytes accounted for a larger fraction of recovered cells in AD, most prominently in Treat_AD3, which alone contributed the largest keratinocyte pool; this pattern is consistent with the epidermal hyperplasia of lesional skin but may also reflect differences in biopsy depth and tissue dissociation. By contrast, endothelial cells generally represented a smaller fraction of recovered cells in AD samples than in healthy samples (Figure 1G), so the data do not support an increase in endothelial abundance in AD. Recovered single-cell proportions reflect tissue sampling and dissociation efficiency in addition to tissue composition. T cells tended to represent a larger fraction of recovered cells in AD samples, macrophage fractions varied between AD donors, and melanocytes were rare in both groups. The pronounced inter-donor variability within the AD group (Figure 1G) may reflect differences in lesional stage, treatment status or immune polarization, which could not be examined further without donor-level clinical data.

### 3.2. Inferred Intercellular Communication in AD

**Figure 2:**
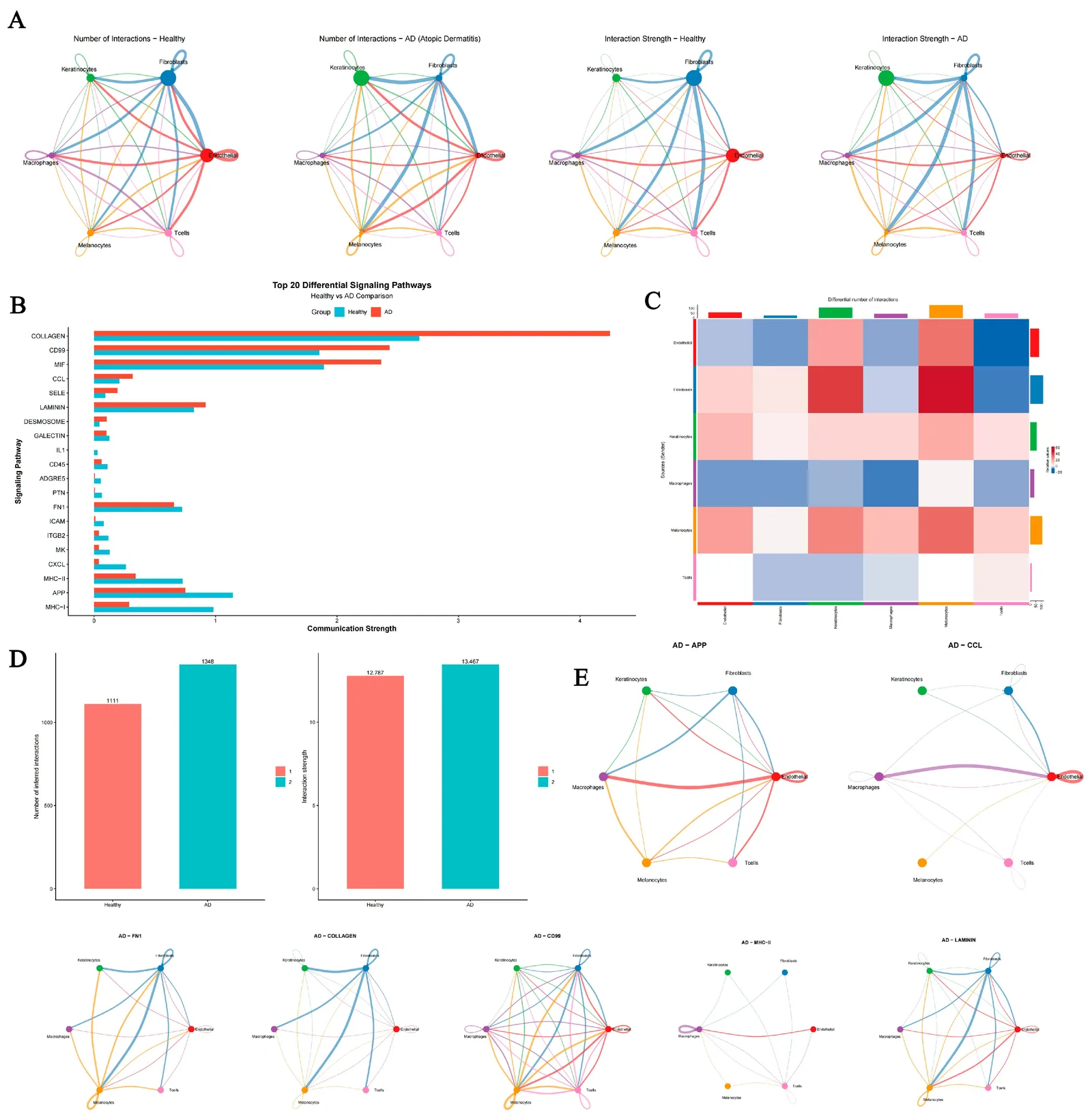
CellChat inference of intercellular communication in healthy and AD skin. (**A**) Chord diagrams comparing the number of interactions and interaction strength between healthy and AD skin across all cell types. (**B**) Communication strength of the top 20 signaling pathways ranked by the difference between healthy and AD skin; COLLAGEN, CD99 and MIF show the largest increases in AD, whereas APP, MHC-I, MHC-II, FN1 and CXCL are higher in healthy skin. (**C**) Heatmap of differential numbers of inferred interactions between sender (rows) and receiver (columns) cell types (AD minus healthy; red, higher in AD; blue, lower in AD). (**D**) Total number of inferred interactions (healthy: 1111; AD: 1348) and aggregate interaction strength, defined as the summed communication probability of all inferred interactions (healthy: 12.787; AD: 13.467). (**E**) Circle plots of pathway-specific inferred communication networks in AD for the APP, CCL, FN1, COLLAGEN, CD99, MHC-II and LAMININ pathways.

Running CellChat separately on the AD and healthy subsets yielded a higher total number of inferred interactions in AD (1348 versus 1111) and a slightly higher aggregate interaction strength (13.467 versus 12.787; Figure 2D). The circle plots (Figure 2A) and the differential heatmap (Figure 2C) indicated a redistribution of inferred interactions among cell types. With endothelial cells as senders, the number of inferred outgoing interactions in AD was higher toward keratinocytes and melanocytes and lower toward T cells, fibroblasts and macrophages (Figure 2C). Endothelial cells, therefore, participated in a reorganized pattern of predicted signaling in AD skin; because CellChat infers ligand–receptor interactions from transcript abundance, these represent candidate signaling routes.

Pathway-level comparison ranked COLLAGEN as the pathway with the largest increase in communication strength in AD (Figure 2B), followed by CD99 and MIF. CCL, SELE, LAMININ and DESMOSOME also showed higher inferred strength in AD, whereas FN1, APP, MHC-I, MHC-II and CXCL were higher in healthy skin (Figure 2B). The sender–receiver heatmap (Figure 2C) showed that endothelial cells and fibroblasts underwent the most pronounced changes in inferred outgoing interactions. Pathway-resolved circle plots for APP, CCL, CD99, COLLAGEN, FN1, LAMININ and MHC-II in AD (Figure 2E) illustrate the cell types predicted to participate in each pathway. Together, these inferred changes point to extracellular matrix-related and adhesion-related signaling as candidate features of the AD vascular niche.

### 3.3. Scoring of AD-Associated Genes Across Skin Cell Types

**Figure 3:**
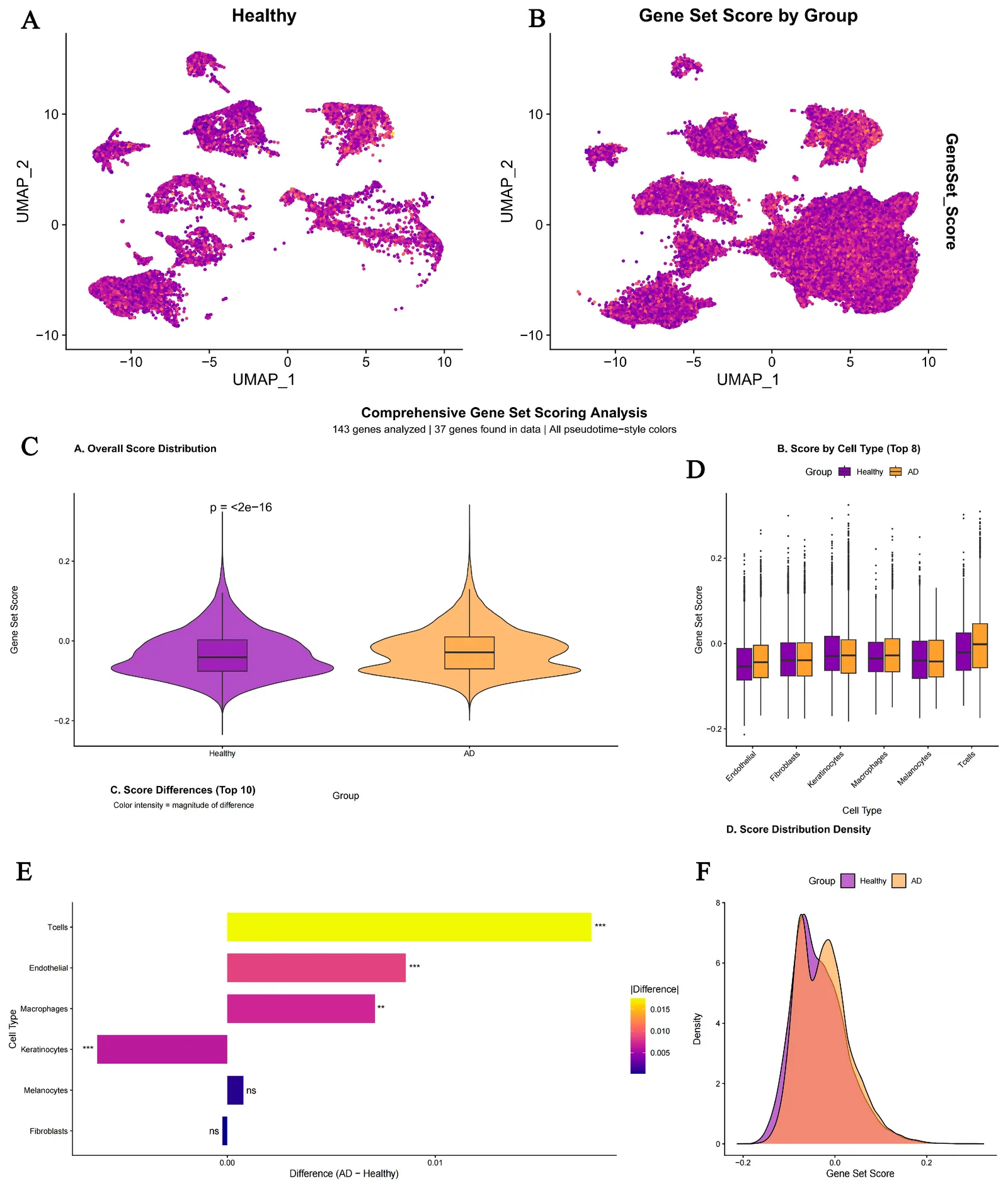
Scoring of an AD-associated gene set across skin cell types. (**A**,**B**) UMAP visualization of AD-associated gene set scores (37 detected genes) in healthy (**A**) and AD (**B**) cells. (**C**) Violin plot of the overall score distribution in healthy and AD cells (cell-level Wilcoxon test, *p* < 2 × 10^−16^). (**D**) Box plots of gene set scores stratified by cell type and group; median scores are higher in AD for T cells, endothelial cells and macrophages, and lower in AD for keratinocytes. (**E**) Score differences (AD minus healthy) per cell type; T cells show the largest increase, followed by endothelial cells and macrophages, keratinocytes show a decrease, and melanocytes and fibroblasts show no significant change (cell-level Wilcoxon tests; **, *p* < 0.01; ***, *p* < 0.001; ns, not significant). (**F**) Kernel density plot of gene set score distributions for healthy and AD cells.

Of the 134 genes in the AD-associated gene set, 37 were detected in the integrated scRNA-seq dataset and were used to compute single-cell gene set scores (Supplementary Table S2). When projected onto UMAP space, the scores were heterogeneous in both conditions, with higher-scoring cells present in several cluster regions (Figure 3A,B).

A global comparison showed higher scores in AD than in healthy cells (cell-level Wilcoxon *p* < 2 × 10^−16^; Figure 3C), although the difference between group medians was small and the *p* value largely reflects the number of cells. Cell-type-stratified comparisons (Figure 3D,E) showed small positive differences in AD for T cells (difference ≈ 0.018), endothelial cells and macrophages (cell-level *p* < 0.01), a small decrease in keratinocytes (cell-level *p* < 0.001), and no significant differences in fibroblasts or melanocytes. The keratinocyte decrease most likely reflects the composition of the gene set, which captures inflammatory and vascular programs more than epidermal barrier biology. The density plot (Figure 3F) showed a bimodal score distribution in both groups, with a relatively larger higher-scoring component in AD cells, which may partly reflect differences in cell-type composition between groups.

### 3.4. hdWGCNA Identifies Seven Endothelial Co-Expression Modules

**Figure 4:**
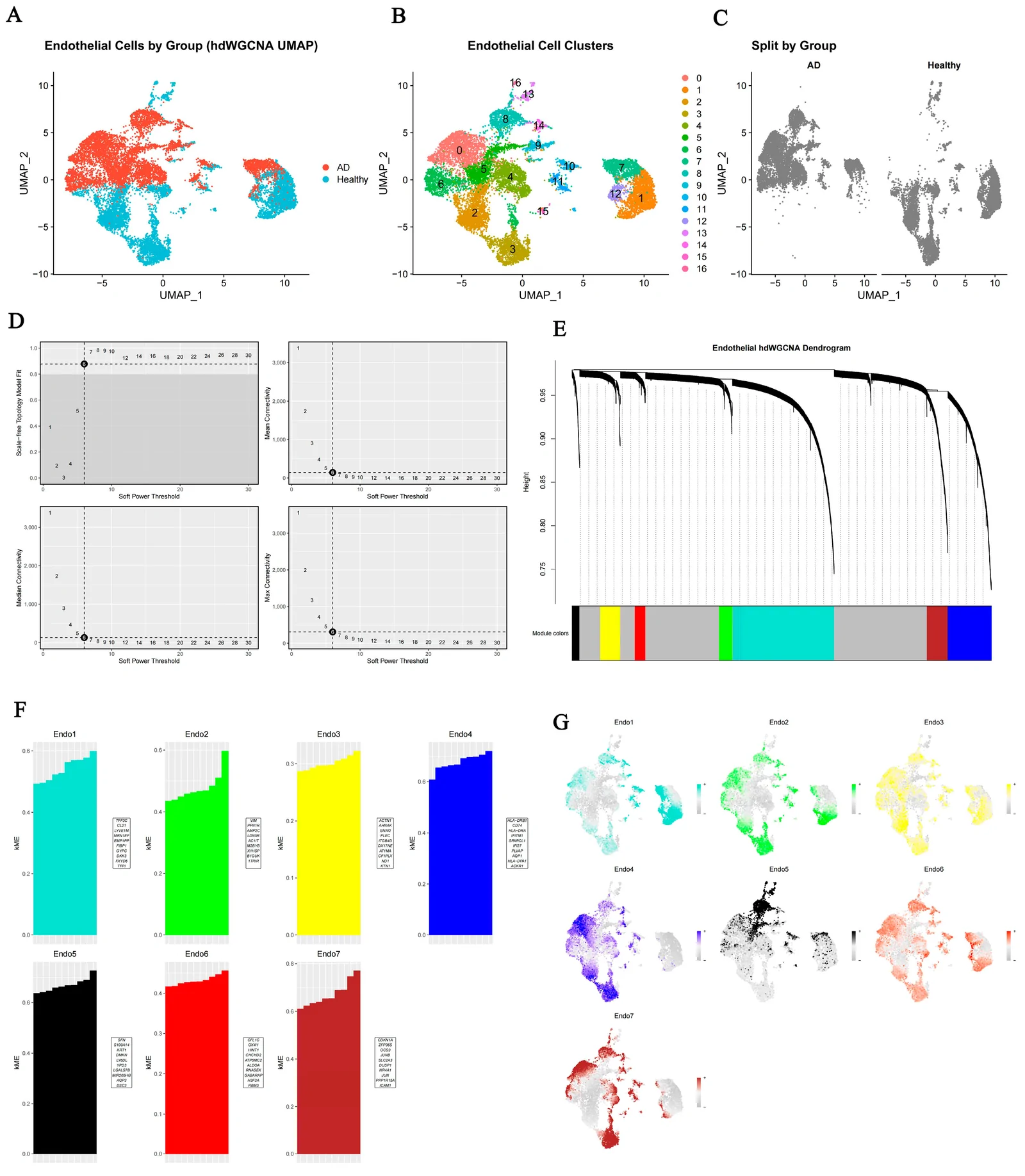
hdWGCNA identifies seven co-expression modules in endothelial cells from AD and healthy skin. (**A**) UMAP of endothelial cells colored by disease group (AD: red; healthy: blue), showing distinct spatial distributions. (**B**) UMAP of endothelial cell sub-clusters (clusters 0–16). (**C**) Split UMAP comparing AD and healthy endothelial cell distributions. (**D**) Soft-power threshold selection plots evaluating scale-free topology model fit, mean connectivity, median connectivity, and maximum connectivity; soft power = 6 was selected. (**E**) Hierarchical dendrogram of endothelial genes with module color assignments, yielding seven modules (Endo1–Endo7). Turquoise, green, yellow, blue, black, red, and brown represent the co-expression modules Endo1–Endo7, respectively, while grey indicates genes not assigned to any module. (**F**) kME values of the ten highest-ranked genes in each module (full lists in Supplementary Table S3). (**G**) UMAP feature plots showing module eigengene scores for Endo1 through Endo7 across the endothelial cell landscape.

Given the gene set scoring results and the CellChat-inferred reorganization of endothelial cell communication, downstream analyses focused on this population. UMAP visualization of the subsetted endothelial cells confirmed that AD and healthy cells occupied partially overlapping but distinct transcriptional territories, with AD cells forming a denser and more spatially extended subcluster along one arm of the embedding (Figure 4A,C). Seventeen sub-clusters were identified within this population (Figure 4B), indicating substantial intra-population heterogeneity. The endothelial subset comprised 13,632 cells (AD, 6978; healthy, 6654). Because the relative abundance of these sub-clusters differed between AD and healthy samples, group-level differences in endothelial gene expression may partly reflect differences in endothelial subtype composition, for example, between blood and lymphatic endothelial cells, rather than uniform changes within each subtype.

Soft power analysis identified β = 6 as the optimal threshold for achieving scale-free network topology (Figure 4D), and hierarchical clustering of the resulting dendrogram delineated seven co-expression modules (Figure 4E). Genes were ranked by kME within each module; the top 100 genes per module and their kME values are listed in Supplementary Table S3, and the ten highest-ranked genes are shown in Figure 4F. Endo1 (cyan) was defined by lymphatic endothelial genes, including TFF3, CCL21, LYVE1 and MMRN1; Endo2 (green) by general endothelial and cytoskeletal genes, including VIM, PFN1, RAMP2 and CLDN5; Endo3 (yellow) by structural and adhesion-related genes, including ACTN1, AHNAK, PLEC and ITGB4; Endo4 (blue) by antigen-presentation and venular endothelial genes, including HLA-DRB1, CD74, HLA-DRA, PLVAP and ACKR1; Endo5 (black) by keratinocyte-associated transcripts, including SFN, S100A14, KRT1 and DMKN, which most likely reflect ambient RNA or residual keratinocyte–endothelial doublets rather than an endothelial program; Endo6 (red) by housekeeping and metabolic genes, including CFL1, COX4I1 and ALDOA; and Endo7 (dark red) by immediate-early and NF-κB-responsive genes, including CDKN1A, ZFP36, SOCS3, JUNB, DUSP1, NR4A1, JUN, ICAM1, IL6, IRF1, NFKBIA and SELE. The highest kME values within each module ranged from 0.32 (Endo3) to 0.77 (Endo7), indicating moderate intramodular connectivity. UMAP projections of module eigengene scores showed that the modules were concentrated in partly distinct regions of the endothelial embedding (Figure 4G).

### 3.5. Endo7 Shows the Strongest Overlap with the AD-Associated Gene Set

**Figure 5:**
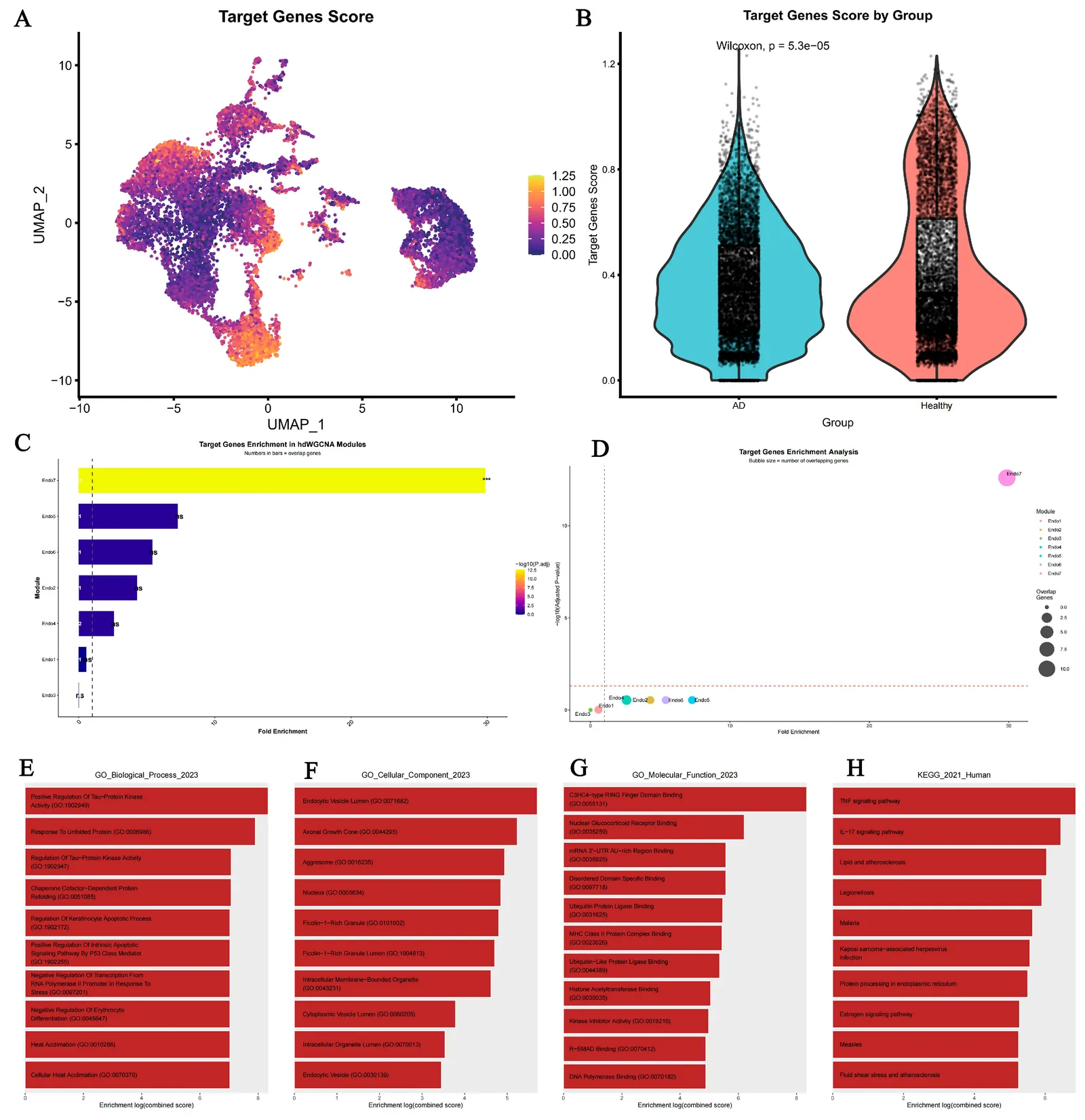
Overlap of the AD-associated gene set with endothelial co-expression modules. (**A**) UMAP of endothelial cells colored by AD target gene set score. (**B**) Violin plot comparing target gene scores between AD (red) and healthy (blue) endothelial cells (cell-level Wilcoxon *p* = 5.3 × 10^−5^). (**C**) Fold enrichment of the AD-associated gene set in the seven hdWGCNA modules; numbers in bars indicate overlapping genes (***, Benjamini–Hochberg adjusted *p* < 0.001; ns, not significant). (**D**) Fold enrichment versus −log10 adjusted *p* for all modules; bubble size indicates the number of overlapping genes. (**E**–**H**) Enrichr analysis of the top 100 Endo7 hub genes against GO Biological Process (**E**), GO Cellular Component (**F**), GO Molecular Function (**G**) and KEGG 2021 Human (**H**) libraries.

When the 37 scored genes of the AD-associated gene set were intersected with the hdWGCNA modules, Endo7 showed the strongest over-representation, with 10 overlapping genes and a fold enrichment of approximately 30 (Benjamini–Hochberg adjusted *p* < 0.001) (Figure 5C,D); the other modules each contained one or two genes of the set and were not significantly enriched. Within the endothelial subset (6978 AD and 6654 healthy cells), target gene scores differed only slightly between groups (Figure 5B): the median score was marginally higher in AD cells (0.348 vs. 0.327), whereas the mean was lower (0.365 vs. 0.409; Cohen’s d = −0.17; cell-level Wilcoxon *p* = 5.3 × 10^−5^), and high-scoring cells were more frequent among healthy cells (29.3% vs. 20.9% of cells in the upper score category). Scores varied considerably between endothelial sub-clusters and were highest in sub-cluster 3 (mean 0.78; Figure 5A), which consisted predominantly of cells from healthy donors (Figure 4A–C). The two distributions, therefore, overlapped extensively.

Functional enrichment of the Endo7 hub genes (Supplementary Table S3) returned GO Biological Process terms including positive regulation of tau-protein kinase activity, response to unfolded protein, chaperone cofactor-dependent protein refolding, heat acclimation and negative regulation of erythrocyte differentiation (Figure 5E). Cellular Component terms included endocytic vesicle lumen, axonal growth cone, aggresome and nucleus (Figure 5F), and Molecular Function terms included C3HC4-type RING finger domain binding, nuclear glucocorticoid receptor binding, MHC class II protein complex binding, ubiquitin-like protein ligase binding and kinase inhibitor activity (Figure 5G). The chaperone- and heat shock-related terms are driven largely by heat shock protein genes such as HSPA1A, HSPA1B and DNAJB1, which also respond to cellular stress during tissue dissociation. Broader terms, such as those related to tau-protein kinase activity or axonal growth cones, arise from shared annotations of a small number of genes.

In the KEGG analysis, TNF signaling and IL-17 signaling pathways ranked highest, followed by lipid and atherosclerosis, legionellosis and fluid shear stress and atherosclerosis (Figure 5H). These terms share NF-κB- and AP-1-dependent inflammatory genes, such as NFKBIA, TNFAIP3, IL6, SOCS3, FOS, JUN, CCL2, ICAM1 and SELE, and point to an endothelial response to inflammatory cytokines, which can be tested at the protein and functional levels.

### 3.6. Distribution of Endothelial Cells Along an Inferred Pseudotime Trajectory

**Figure 6:**
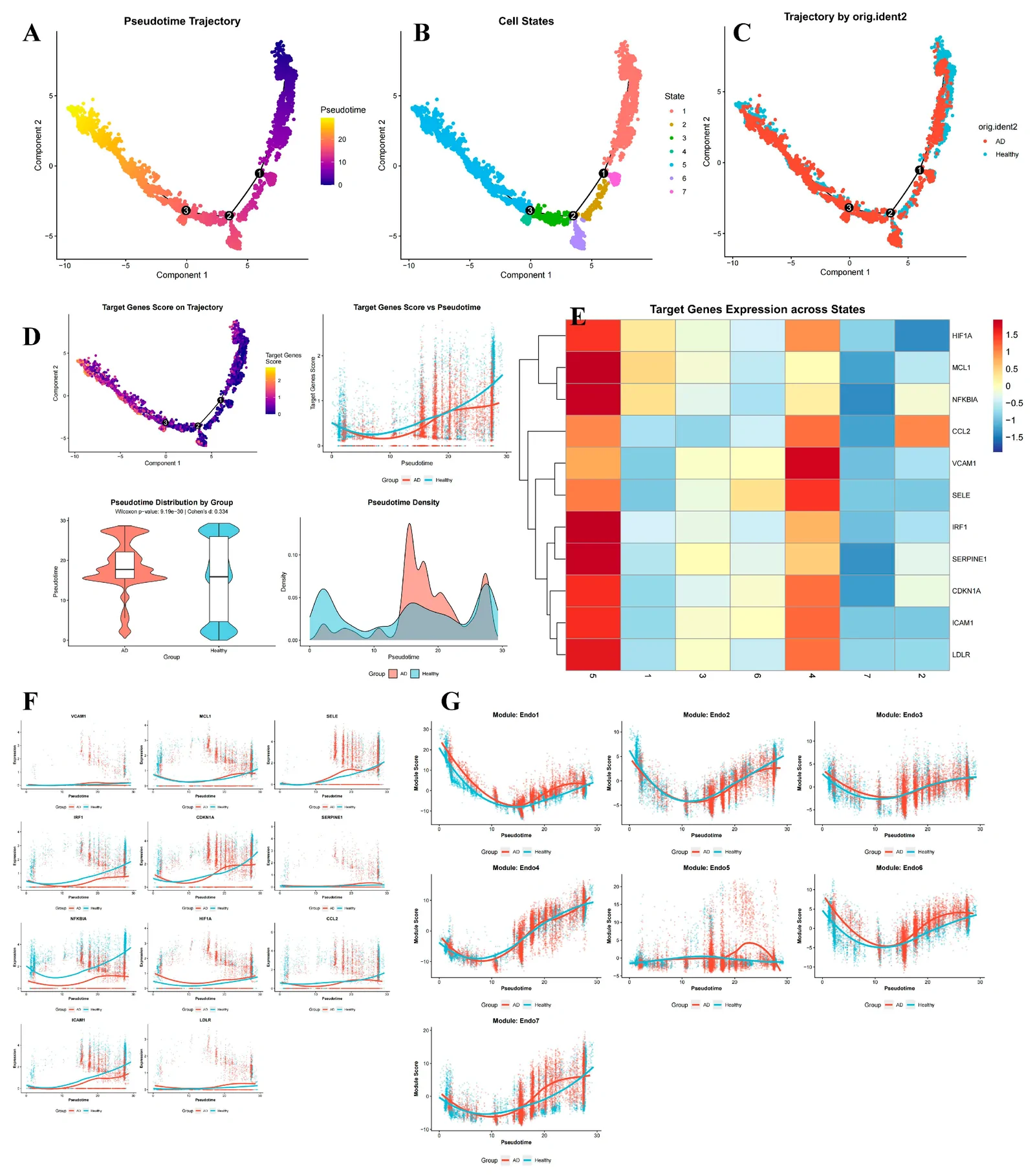
Pseudotime ordering of endothelial cells from AD and healthy skin. (**A**) Monocle 2 pseudotime trajectory colored by pseudotime value (0–30); numbered circles indicate branch points. (**B**) Trajectory colored by inferred cell state (states 1–7). (**C**) Trajectory colored by group (AD: red; healthy: blue). (**D**) Target gene set score overlaid on the trajectory (**top left**); target gene score versus pseudotime by group with smoothed fits (**top right**); violin plot of pseudotime by group (cell-level Wilcoxon *p* = 9.19 × 10^−30^; Cohen’s d = 0.334; **bottom left**); pseudotime density curves by group (**bottom right**). (**E**) Heatmap of scaled expression of HIF1A, MCL1, NFKBIA, CCL2, VCAM1, SELE, IRF1, SERPINE1, CDKN1A, ICAM1 and LDLR across cell states. (**F**) Expression of the same genes along pseudotime, with locally smoothed fits stratified by group. (**G**) Module eigengene scores for Endo1–Endo7 along pseudotime by group (AD: red; healthy: blue).

Monocle 2 ordered endothelial cells along a principal graph with three branch points and seven cell states (Figure 6A,B); the root was placed at the end of state 1, the state with the highest proportion of cells from healthy donors. Healthy endothelial cells were located toward both ends of the trajectory, whereas AD cells were concentrated in the intermediate segment between branch points 2 and 3 (Figure 6C). This separation was partial, and cells from both groups were present along the whole trajectory.

Median pseudotime was higher in AD than in healthy endothelial cells (cell-level Wilcoxon *p* = 9.19 × 10^−30^; Cohen’s d = 0.334; Figure 6D), a small-to-moderate effect by conventional benchmarks. The density plots showed that this difference arose mainly from the concentration of AD cells at intermediate pseudotime values (approximately 15–22), whereas healthy cells showed peaks near both the beginning and the end of the trajectory; cells from both groups were present at late pseudotime. Target gene scores tended to increase with pseudotime in both groups, and the fitted curve for healthy cells was higher than that for AD cells in the latest interval (Figure 6D).

The state-resolved expression heatmap (Figure 6E) showed that most of the displayed genes, including HIF1A, MCL1, NFKBIA, IRF1, SERPINE1, CDKN1A, ICAM1 and LDLR, had their highest scaled expression in state 5, located at the distal end of the trajectory, whereas VCAM1, SELE and CCL2 were highest in state 4, adjacent to branch point 3; the expression of most genes was lowest in state 7. In the gene-level trajectory plots (Figure 6F), fitted curves for AD and healthy cells overlapped for several genes, and the direction of group differences was not uniform: SELE showed higher fitted expression in AD cells at intermediate pseudotime, whereas NFKBIA, IRF1, CDKN1A and ICAM1 showed higher fitted expression in healthy cells over much of the trajectory. Among module eigengene trajectories (Figure 6G), Endo7 scores were higher in AD cells at intermediate pseudotime (approximately 15–25) and converged with those of healthy cells at the terminal end, and Endo5 showed an AD-specific increase in the same interval, whereas the curves for the remaining modules largely overlapped.

### 3.7. Machine Learning and SHAP Analysis

**Figure 7:**
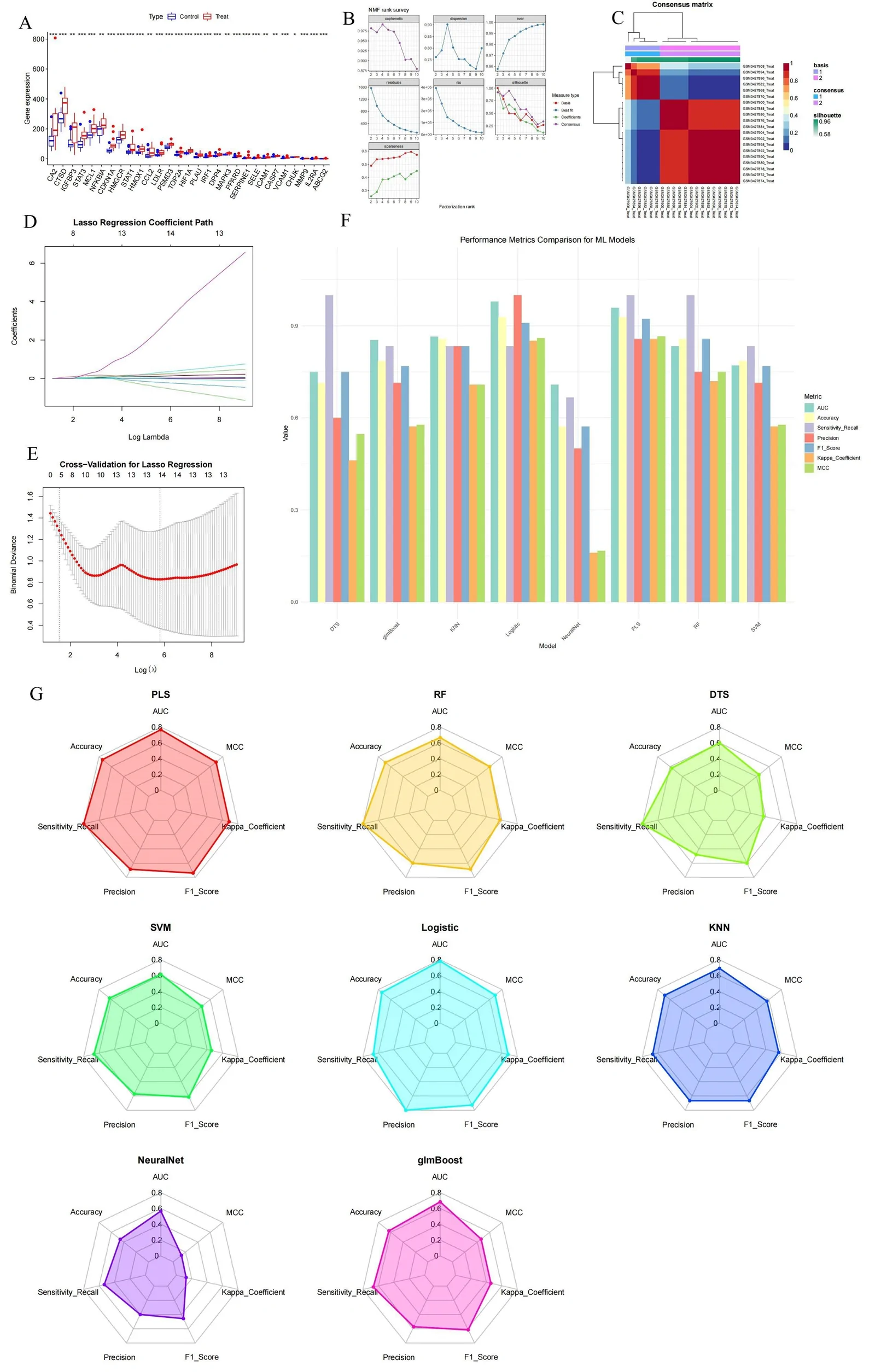
Machine learning classification of lesional and non-lesional AD skin in a bulk transcriptomic dataset. (**A**) Box plots of candidate gene expression in non-lesional (Control) and lesional (Treat) AD skin samples from the bulk dataset (GSE121212). (**B**) NMF rank survey across ranks 2–10 (cophenetic, dispersion, silhouette, sparseness and residual metrics). (**C**) Consensus matrix of the lesional samples at rank 2. (**D**) LASSO regression coefficient path plot showing variable selection across log(λ) values. (**E**) Cross-validation curve for LASSO regression identifying optimal λ by minimum binomial deviance. (**F**) Test-set performance metrics (AUC, accuracy, sensitivity/recall, precision, F1 score, Kappa coefficient, MCC) of the eight classifiers (n = 14 test samples). (**G**) Radar plots of the seven performance metrics for each classifier.

In the bulk dataset, the candidate genes shown in Figure 7A, including SELE, VCAM1, IRF1, CASP7 and CCL2, differed significantly between lesional and non-lesional AD skin. NMF of the lesional samples favored a rank of 2 (Figure 7B), and the consensus matrix separated these samples into two clusters, with silhouette widths ranging from 0.58 to 0.96 (Figure 7C). LASSO regression with cross-validated lambda selection retained 14 informative features at the optimal penalty parameter, as shown by the coefficient path and cross-validation deviance curves (Figure 7D,E).

In the held-out test set (n = 14; 6 lesional and 8 non-lesional samples), AUC values ranged from approximately 0.70 to 0.98 (likely optimistic estimates, as LASSO feature selection preceded the training–test split and paired lesional and non-lesional samples from the same patients may span both partitions; Figure 7F): logistic regression at 0.98, PLS-DA at 0.96, KNN at 0.86, glmBoost at 0.85, RF at 0.83, SVM at 0.77, DTS at 0.75 and the neural network at 0.70. Logistic regression and PLS-DA each classified 13 of the 14 test samples correctly (accuracy 0.93; exact binomial 95% CI 0.66–1.00), whereas the neural network showed little agreement beyond chance (Cohen’s Kappa and MCC below 0.2). Radar charts of the seven evaluation metrics (Figure 7G) showed that performance varied considerably between algorithms. With 14 test samples, these estimates carry considerable uncertainty.

**Figure 8:**
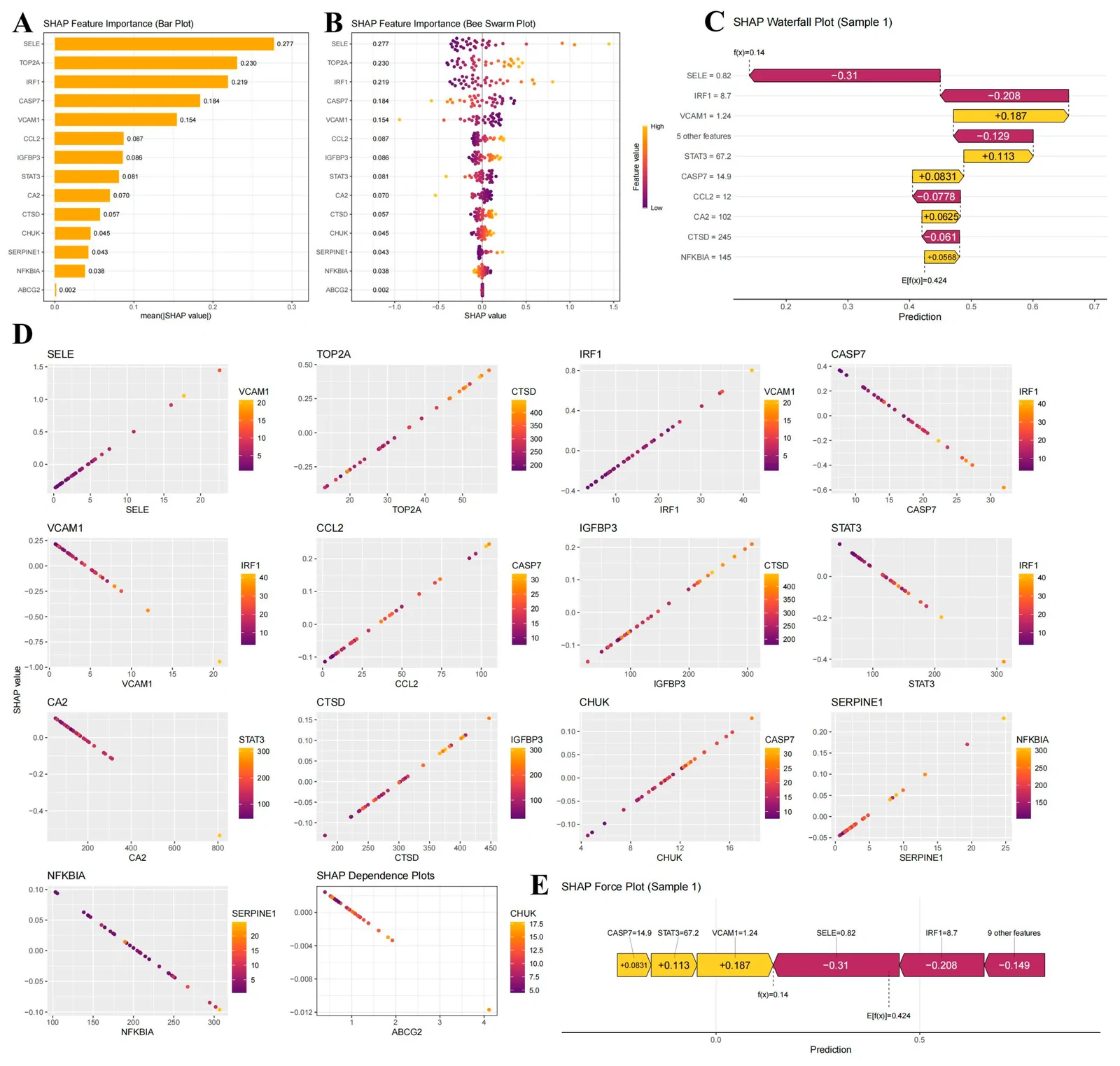
SHAP interpretation of the logistic regression classifier. (**A**) SHAP feature importance bar plot ranking predictive genes by mean |SHAP| value; SELE (0.277), TOP2A (0.230), and IRF1 (0.219) rank as the top three features. (**B**) SHAP beeswarm plot showing the distribution and directionality of SHAP values for each feature across samples. (**C**) SHAP waterfall plot for a representative sample illustrating individual feature contributions to the model prediction; SELE and IRF1 exert the largest negative contributions while VCAM1 contributes positively. (**D**) SHAP dependence plots for all features; the color scale shows the value of a second feature. (**E**) SHAP force plot for the same representative sample summarizing the composite push of features toward a higher or lower predicted probability of lesional skin.

SHAP analysis of the logistic regression model ranked SELE as the most influential feature (mean |SHAP| = 0.277), followed by TOP2A (0.230) and IRF1 (0.219) (Figure 8A). In the beeswarm plot, higher SELE expression was associated with positive SHAP values, that is, with a higher predicted probability that a sample was lesional (Figure 8B). The waterfall and force plots for one representative sample (Figure 8C,E) illustrated how individual contributions combined: in this sample, low SELE (0.82) and IRF1 (8.7) values contributed −0.31 and −0.208, respectively, lowering the predicted probability from the expected value E[f(x)] = 0.424, which equals the proportion of lesional samples in the training set (14/33), to f(x) = 0.14, whereas VCAM1 (1.24) contributed +0.187. The dependence plots (Figure 8D) showed approximately linear relationships between each feature value and its SHAP value, consistent with the linear predictor of the logistic regression model, and the color scale shows the value of a second feature. VCAM1, CASP7 and STAT3 showed negative slopes, meaning that, conditional on the other features in the model, higher values lowered the predicted probability of lesional skin. Such negative contributions can arise from correlation among features in a multivariable model.

## 4. Discussion

AD has long been framed as an immune–epithelial disease, with therapeutic development concentrated on the Th2/Th17 axis and epidermal barrier dysfunction [56,57]. The vascular compartment receives comparatively little attention, despite the clinical visibility of erythema, edema, and leukocyte infiltration—all of which are obligatorily vascular events. In this exploratory reanalysis, complementary analyses of a single-cell dataset pointed to a coordinated endothelial activation signature in AD skin. Because these analyses derive from one dataset, independent cohorts will be needed to determine whether this signature reflects an intrinsic endothelial program or a response to the inflammatory milieu. The composition of the Endo7 module and previous histological studies [17,18] favor the latter, and activated endothelium may, in turn, contribute to leukocyte recruitment.

Our analyses provide evidence of differing strength that endothelial cells are transcriptionally engaged in AD lesions. Gene set scoring provided only weak evidence: in the whole-dataset analysis, endothelial cells showed a small increase in AD-associated scores, together with T cells and macrophages, but this difference was not reproduced within the endothelial subset, where the mean score was slightly lower in AD cells (Cohen’s d = −0.17). More informative were two further observations. First, CellChat inferred a redistribution of endothelial interactions in AD, with more predicted outgoing interactions toward keratinocytes and melanocytes and fewer toward T cells, fibroblasts and macrophages. Second, the Endo7 module, which showed the strongest overlap with the AD-associated gene set, comprised a coordinately expressed group of NF-κB-responsive and immediate-early genes, including SELE, ICAM1, IRF1, NFKBIA, IL6 and TNFAIP3. These are classical targets of TNF-α and IL-1β signaling in endothelial cells [15,58,59], and the enrichment of TNF and IL-17 signaling pathways in this module is consistent with cytokine-induced activation. Cytokine-driven activation is, therefore, a likely explanation for this program. Several of the highest-ranked Endo7 genes, including FOS, JUN, JUNB, NR4A1, DUSP1, HSPA1A and HSPA1B, are also induced by enzymatic tissue dissociation [60], which may also contribute to this module. An expansion of activated post-capillary venule endothelial cells has been described in erythrodermic psoriasis [61], and prenatal vascular programs re-emerge in inflammatory skin diseases [24], suggesting that endothelial activation may be a shared feature of chronic cutaneous inflammation.

Several genes highlighted by our analyses have established roles in vascular inflammation. SELE and VCAM1 are canonical endothelial activation markers whose induction by TNF-α and IL-1β has been well characterized in vitro [58,59]. In our data, VCAM1 transcripts were detected at low levels in endothelial cells from both groups, in line with the absence of endothelial VCAM-1 staining reported in a recent immunohistochemical study [18]. HIF1A and SERPINE1 were among the genes whose expression varied along the endothelial trajectory (Figure 6E), but their differences between AD and healthy endothelial cells were small and neither ranked among the top Endo7 hub genes. Because HIF-1α is regulated predominantly at the protein level through oxygen-dependent proteasomal degradation, HIF1A transcript levels alone do not reveal HIF-1α activity [62,63,64]. Inflammatory mediators can stabilize HIF-1α under normoxic conditions in other inflammatory skin diseases [65,66,67,68], and whether this occurs in AD endothelium is an open question. Similarly, a prothrombotic endothelial phenotype, described mainly in psoriasis and systemic lupus erythematosus [69,70,71,72,73,74], would need to be established at the protein and functional levels. Hypoxia-like reprogramming and prothrombotic activation of AD endothelium can be tested by HIF-1α immunostaining, measurement of PAI-1 protein and functional assays in cytokine-stimulated dermal endothelial cells.

In the pseudotime analysis, AD endothelial cells were concentrated at intermediate positions, whereas healthy cells were distributed toward both ends of the trajectory, with a small-to-moderate overall effect (Cohen’s d = 0.334). Because the analysis is cross-sectional and its direction depends on the choice of root, questions of reversibility, commitment or a therapeutic window will require longitudinal sampling, lineage tracing or perturbation experiments. NFKBIA expression tended to increase along pseudotime, particularly in healthy cells, and was lower overall in AD than in healthy endothelial cells (Figure 6F; Supplementary Table S4). Because IκBα is itself encoded by an NF-κB target gene, NFKBIA expression is best interpreted as a marker of NF-κB activation accompanied by inducible negative feedback [75,76]. Whether endothelial activation persists during systemic treatment is an open question that will require treatment-related data.

The second-ranked SHAP predictor, TOP2A, encodes DNA topoisomerase II alpha, a marker canonically associated with cellular proliferation and DNA replication stress [77]. TOP2A did not rank among the top endothelial hub genes in our co-expression analysis, and because the classifier was trained on bulk transcriptomes of lesional and non-lesional skin, its contribution most likely reflects proliferating keratinocytes and immune cells in lesional skin. Endothelial proliferation in inflammatory skin disease has been documented in psoriasis and wound healing contexts [78,79], and whether a subset of AD endothelial cells is cycling is a hypothesis that merits direct testing, for example, with Ki-67 and CD31 co-staining of lesional tissue. IRF1 is a primary transcriptional mediator of IFN-γ signaling [80]. Chronic AD lesions are characterized by progressive activation of Th2 and Th22 pathways, with additional Th1 and Th17 components that are superimposed on type 2 inflammation [10,81]. In our single-cell data, IRF1 belonged to the Endo7 module but was expressed at lower levels in AD than in healthy endothelial cells, and IRF1 is expressed by many cell types. Whether interferon–IRF1 signaling, and its possible link to CASP7-associated cell death suggested by the classifier, operates in AD endothelium is, therefore, a question for experimental investigation.

In summary, our findings describe an AD-associated endothelial activation signature, dominated by NF-κB-responsive and immediate-early genes, that is embedded in a reorganized network of inferred intercellular communication. Adhesion molecule blockade, modulation of HIF-1α signaling and interference with interferon–IRF1 signaling are candidate strategies for experimental investigation in AD, and whether endothelial-directed approaches could complement biologics targeting IL-4Rα, IL-13 or IL-31 remains an open question. Experimental AD models, which have been used to evaluate a range of anti-inflammatory candidates [11,12], together with cytokine-stimulated dermal endothelial cells, would provide suitable systems for testing these hypotheses.

## 5. Limitations

Several limitations should be considered when interpreting these findings. First, the single-cell analysis included only nine donors (five with AD and four healthy controls) from a single public dataset, which limits statistical power and generalizability. Individual-level clinical information, including age, sex, disease severity, disease duration, treatment exposure and sampling characteristics, was not available for the AD donors (Supplementary Table S1). Therefore, we could not assess confounding by these factors or determine whether the findings apply equally to children and adults, or to different disease stages and endotypes. Second, cells from the same donor are not independent biological replicates. Most statistical comparisons in this study were performed at the cell level, which can produce very small *p* values and overstate the strength of evidence [82,83]; donor-level pseudobulk or mixed-effects analyses could not be performed robustly with four to five donors per group and will be required in larger cohorts. Third, the machine learning analysis was based on bulk skin transcriptomes and compared lesional with non-lesional skin from patients with AD, so the selected features reflect tissue-level differences between lesional and non-lesional skin to which many cell types contribute; they cannot be attributed specifically to endothelial cells and do not address the distinction between AD and healthy skin. The held-out test set was small, LASSO feature selection preceded the training–test split, paired lesional and non-lesional samples from the same patients may have been allocated to different partitions, and no external validation was performed; performance estimates are, therefore, likely to be optimistic, and SELE, TOP2A and IRF1 should be regarded as candidate discriminatory genes and not as validated biomarkers. Fourth, the AD-associated gene set was derived from database annotations and compound–target predictions, is biased toward well-annotated, pharmacologically targetable proteins, and only 37 of its 134 genes could be scored. Moreover, gene set scores, CellChat predictions and co-expression modules were all derived from transcript abundance, and donor effects were not explicitly modeled in the co-expression network; these analyses do not establish protein expression, receptor activation or signaling directionality, and their consistency across analyses of the same dataset does not constitute independent validation. Fifth, pseudotime trajectories inferred from cross-sectional data do not establish temporal progression, reversibility or commitment, and their direction depends on the choice of root. Sixth, the endothelial transcriptome may be affected by technical factors. Keratinocyte-derived transcripts, such as S100A8, S100A9, KRT6A and KRT16, were detected in endothelial cells, particularly in AD samples, and defined the Endo5 module, suggesting ambient RNA contamination or residual doublets [84]; in addition, several Endo7 genes are induced by enzymatic tissue dissociation [60]. Differences in endothelial subtype composition between AD and healthy samples, such as the relative abundance of lymphatic (Endo1) and venular (Endo4) endothelial cells, may also contribute to group-level expression differences. Finally, no independent single-cell cohort, spatial transcriptomic, immunohistochemical or functional validation was performed. The present study should, therefore, be regarded as a hypothesis-generating analysis. In future work, we plan to validate these findings in independent, clinically characterized cohorts, to confirm the expression of candidate proteins such as E-selectin, ICAM-1 and IRF1 in lesional endothelium in situ, and to perform functional experiments in dermal endothelial cells.

## 6. Conclusions

This exploratory reanalysis identifies AD-associated endothelial transcriptional signatures and candidate genes for further investigation. In AD skin, endothelial cells participated in a reorganized network of inferred intercellular communication and contained an NF-κB-responsive co-expression module (Endo7) that overlapped with AD-associated genes, including SELE, ICAM1 and IRF1, whereas differences in overall AD-associated gene set scores between AD and healthy endothelial cells were small and inconsistent. These observations are consistent across complementary analyses of a single dataset. Their genetic relevance, functional significance, and diagnostic or therapeutic utility require confirmation in independent, clinically characterized cohorts and experimental studies.

Future studies should examine whether endothelial activation contributes causally to AD initiation or chronicity, using spatial transcriptomics, immunostaining of lesional skin and perturbation models. Endothelial-directed strategies such as adhesion molecule blockade and circulating markers such as soluble E-selectin remain research questions to be evaluated alongside existing biologics.

## Data Availability

All data are sourced from public databases. Further enquiries can be directed to the corresponding author. The single-cell RNA-sequencing data analyzed in this study are publicly available in the Gene Expression Omnibus database under accession number GSE269981. The bulk RNA-seq data used for the machine learning analysis are available in the Gene Expression Omnibus under accession number GSE121212. AD-related genes were obtained from the publicly accessible GeneCards, OMIM and DisGeNET databases. The analysis scripts, including essential parameters, are available from the corresponding author upon reasonable request.

## Supplementary Materials

The following supporting information can be downloaded at https://www.mdpi.com/article/10.3390/cimb48101039/s1.
